# Is HPV-18 present in human breast cancer cell lines?

**DOI:** 10.1038/sj.bjc.6605671

**Published:** 2010-04-20

**Authors:** I Peran, A Riegel, Y Dai, R Schlegel, X Liu

**Affiliations:** 1 Department of Pathology, Georgetown University Medical Center, Washington, DC, USA; 2 Department of Oncology; Lombardi Comprehensive Cancer Center, Georgetown University Medical Center, Washington, DC, USA

Sir,

Although several studies have suggested an association between breast cancer and human papillomavirus (HPV) infection (de Villiers *et al*, 2005; Cazzaniga *et al*, 2009), two recent papers published in the *British Journal of Cancer* (Heng *et al*, 2009; Lawson *et al*, 2009) were particularly provocative. The authors claimed that both primary human breast cancers and two well-characterised breast cancer cell lines (MDA-MB-175VII and SK-Br-3) contained HPV-18, a type of HPV found with increased frequency in adenocarcinomas (Iwasawa *et al*, 1996; Burk *et al*, 2003).

To further characterise the MDA-MB-175VII and SK-Br-3 cell lines for HPV-18 gene content and expression, we performed PCR and RT–PCR to detect viral DNA and mRNA. We also included two cell lines as controls. The HeLa cervical cancer cell line contains HPV-18 and was used as a positive control and the C33 cervical cancer cell line contains no detectable HPV genomes. All cell lines were grown in 10% FBS DMEM media to approximately 85% confluency and then harvested for DNA and RNA isolation, after which we performed standard PCR and RT–PCR reactions using the indicated primer sets for the early, late, and non-coding regions of HPV-18 (see Figure 1A). For reference, we also used the exact L1 primer sequence set that was used by Heng *et al* for detection of HPV-18 in MDA-MB-175VII and SK-Br-3 by *in situ* PCR.

In order to detect HPV-18 DNA, cellular DNA was isolated using a Qiagen DNA isolation kit. After performing PCR reactions (95°C for 5 min, 35 cycles: 95°C for 30 s, 55°C for 30 s and 72°C for 1 min, with a final extension at 72°C for 5 min) using different sets of primers, the PCR products were separated on a 2% agarose gel. Consistent with the studies of Schneider-Gadicke and Schwarz (1986) that mapped the genomic fragments of HPV-18 present in HeLa cells, our primer sets were able to amplify the L1, NCR, E6 and E7 regions of HPV-18 DNA in HeLa cells (panel B). C33A cells were uniformly negative (panel B). Surprisingly, the MDA-MB-175VII and SK-Br-3 breast cell lines were completely negative for HPV-18 DNA (panel B), indicating either the absence or very low abundance of HPV-18 DNA in these cell lines. As an internal control for verifying DNA quality isolated from the above cell lines, we performed PCR with primers specific for human β globin (HBG). All samples were uniformly positive for the presence of this gene (panel B, bottom).

To address the possibility that the copy number of HPV-18 genomes was extremely low in these cells and undetectable by PCR, we also performed more sensitive RT–PCR reactions to detect HPV-18 mRNA. Cellular RNA was isolated by the TRIzol method, followed by one-step RT–PCR (42°C for 60 min, 95°C for 2 min, 35 cycles: 95°C for 30 s, 55°C for 30 s and 72°C for 1 min, with a final extension at 72°C for 5 min). PCR products were separated on a 2% agarose gel. In HeLa cells we detected transcription products for the two major transforming genes of HPV-18, *E6* and *E7* (panel D). As expected, we also detected the long and short size variants of E6 mRNA that are generated by RNA splicing. Corresponding to our PCR data that indicated a lack of HPV DNA in the breast cells, we also found no evidence for expression of HPV-18 mRNA (panel D). To validate our RNA purification, we performed RT–PCR for GAPDH mRNA, which demonstrated that the RNA samples were of sufficient quality to detect the expression of a single copy gene.

To conclude, HPV-18 DNA and mRNA are not detectable in the MDA-MB-175VII and SK-Br-3 breast cancer cell lines, contradicting the study of Heng *et al.* As the *E6* and *E7* genes of the high-risk HPVs are retained and expressed in all HPV-induced cervical cancers (Androphy *et al*, 1987; Banks *et al*, 1987; Hawley-Nelson *et al*, 1989; Munger and Howley, 2002) and their cooperative interaction is required for efficient cell immortalisation and maintenance of the tumourigenic phenotype (Androphy *et al*, 1987; Banks *et al*, 1987; Hawley-Nelson *et al*, 1989; Munger and Howley, 2002), our results strongly indicate that HPV is not an aetiologic factor in the generation of these breast tumour cell lines. Although there may be a subset of breast cancers that are induced by HPV, the MDA-MB-175VII and SK-Br-3 cell lines clearly cannot be used to support this hypothesis and they are not valid cell lines for studying HPV-mediated transformation of breast cells.

## Figures and Tables

**Figure 1:**
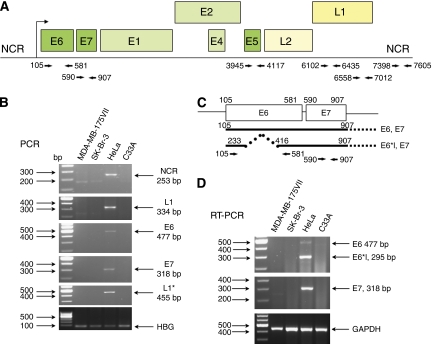
Absence of HPV-18 in the MDA-MB-175VII and SK-Br-3 breast cancer cell lines. (**A**) HPV-18 genome structure and PCR primer sets used in this study. (**B**) PCR for HPV-18 DNA in cell lines. The indicated primer sets were used to amplify the NCR, L1, E6 and E7 regions of HPV18 DNA. L1^*^ indicates the primer set used in the article by Heng *et al.* (**C**) Early transcripts of HPV-18 and primer sets used for RT–PCR. (**D**) RT–PCR for mRNAs of HPV-18 early genes (*E6* and *E7*).
